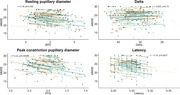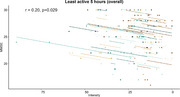# Prognostic and Disease Tracking Capability of Light Reflex Pupillometry and Actigraphy in Early Alzheimer's Disease

**DOI:** 10.1002/alz70856_097029

**Published:** 2025-12-24

**Authors:** Mathias Holsey Gramkow, Frederikke Kragh Clemmensen, Andreas Brink‐Kjær, Ulrich Lindberg, Ian Law, Otto Mølby Henriksen, Gunhild Waldemar, Poul Jørgen Jennum, Steen G. Hasselbalch, Kristian Steen Frederiksen

**Affiliations:** ^1^ Danish Dementia Research Centre, Dept. of Neurology, Copenhagen University Hospital ‐ Rigshospitalet, Copenhagen, Denmark; ^2^ Department of Health Technology, Technical University of Denmark, Kongens Lyngby, Greater Copenhagen, Denmark; ^3^ Department of Clinical Neurophysiology, Danish Center for Sleep Medicine, Copenhagen University Hospital ‐ Rigshospitalet, Copenhagen, Denmark; ^4^ Functional Imaging Unit, Department of Clinical Physiology and Nuclear Medicine, Copenhagen University Hospital ‐ Rigshospitalet, Copenhagen, Copenhagen, Denmark; ^5^ Department of Clinical Medicine, Faculty of Health and Medical Sciences, University of Copenhagen, Copenhagen, Denmark; ^6^ Department of Clinical Physiology and Nuclear Medicine, Copenhagen University Hospital ‐ Rigshospitalet, Copenhagen, Denmark

## Abstract

**Background:**

Easily acquired biomarkers for the prognosis of Alzheimer's disease are crucial in the current paradigm of disease‐modifying therapies. Light reflex pupillometry (qLRP) measures distinct midbrain functions, and actigraphy measures circadian rhythm and activity. Both have been shown to be altered in AD. We aimed to determine the prognostic and disease‐tracking capability of qLRP and actigraphy in early Alzheimer's disease.

**Method:**

In this single‐center longitudinal cohort study, 86 patients with AD (mean age 75.7 years, MMSE 25.8) were included in the pupillometry study and 58 patients (mean age 74.8 years, MMSE 25.5) were included in the actigraphy study and followed for 18‐24 months. qLRP (PLR‐3000, NeurOptics®) and dual sensor, body‐worn actigraphy (SENS® Motion) were performed at baseline and follow‐up visits. Clinical and neuroimaging ([^18^F]‐FDG‐PET visual read) progression and cognitive decline (MMSE) were determined at the 1‐year follow‐up. Logistic regression models were fitted with longitudinal and baseline qLRP and actigraphy features as predictors in separate models (adjusted for age and sex). Repeated measures correlation was used to investigate longitudinal correlations with changes in MMSE.

**Result:**

A decrease from baseline‐3‐months FU in the resting pupillary diameter was associated with higher risk of clinical progression (odds ratio 4.28, 95 % CI: 1.24‐16.9) and showed a significant correlation with cognitive decline during follow‐up (r=0.17, *p* = 0.008) (Figure 1). In addition, baseline relative change in pupil size could predict neuroimaging progression (AUC 0.64, 95 % CI: 0.50‐0.77). Several baseline rest/activity features, including the intra‐daily variability of the number of steps (*p* = 0.016), could predict clinical progression, and lower activity levels during evening and nighttime predicted cognitive decline (delta‐MMSE>‐3) (*p* = 0.01). Longitudinally, decreases in intensity during the least active five hours correlated with decline on the MMSE (r=0.20, *p* = 0.029).

**Conclusion:**

Our results indicate that dynamic pupillary changes were associated with a higher risk of progression, possibly reflecting changes in arousal states. qLRP also correlated with disease progression along the disease continuum. The same pattern was found for several actigraphic parameters, indicating that decreases in nighttime activity were indicative of disease progression. This study demonstrates the usefulness of two promising digital biomarkers for AD easing prognostication and disease tracking.